# Supplementary material for: Structures of Helicobacter pylori C-terminal protease CtpA reveal a new mode of self-contained proteolytic processing
Source: Commun Biol. 2025 Nov 22;8:1828. doi: 10.1038/s42003-025-09175-5 (PMC12749690; doi:10.1038/s42003-025-09175-5)
Supplement: Supplementary file 2 — Supplementary Information [file 42003_2025_9175_MOESM2_ESM.pdf]

Supplementary information

Structures of *Helicobacter pylori* C-terminal protease CtpA reveal a new mode of self-contained proteolytic processing

Kailei Sun[1], Lili Yan[1], Chin Yu Mok[1], Pui Kin So[2], Sirius Pui Kam Tse[2], Kwok Fai Lau[3], Daping Wang[4], Huawei Zhang[5,6,*], Shannon Wing Ngor Au[1,*]

[1]Center for Protein Science and Crystallography, School of Life Sciences, The Chinese University of Hong Kong, Shatin, Hong Kong, China.

[2]State Key Laboratory of Chemical Biology and Drug Discovery, Department of Applied Biology and Chemical Technology, The Hong Kong Polytechnic University, Kowloon, Hong Kong, China.

[3]School of Life Sciences, The Chinese University of Hong Kong, Shatin, Hong Kong, China.

[4]Department of Orthopedics, Shenzhen Intelligent Orthopaedics and Biomedical Innovation Platform, Guangdong Artificial Intelligence Biomedical Innovation Platform, Shenzhen Second People's Hospital, the First Affiliated Hospital of Shenzhen University Health Science Center, Shenzhen, 518035, China.

[5]Shenzhen Institute of Advanced Technology, Chinese Academy of Sciences, 518055, Shenzhen, China.

[6]School of Life Sciences, Southern University of Science and Technology, 518055, Shenzhen, China.

* Co-correspondence: hw.zhang@siat.ac.cn, shannon-au@cuhk.edu.hk

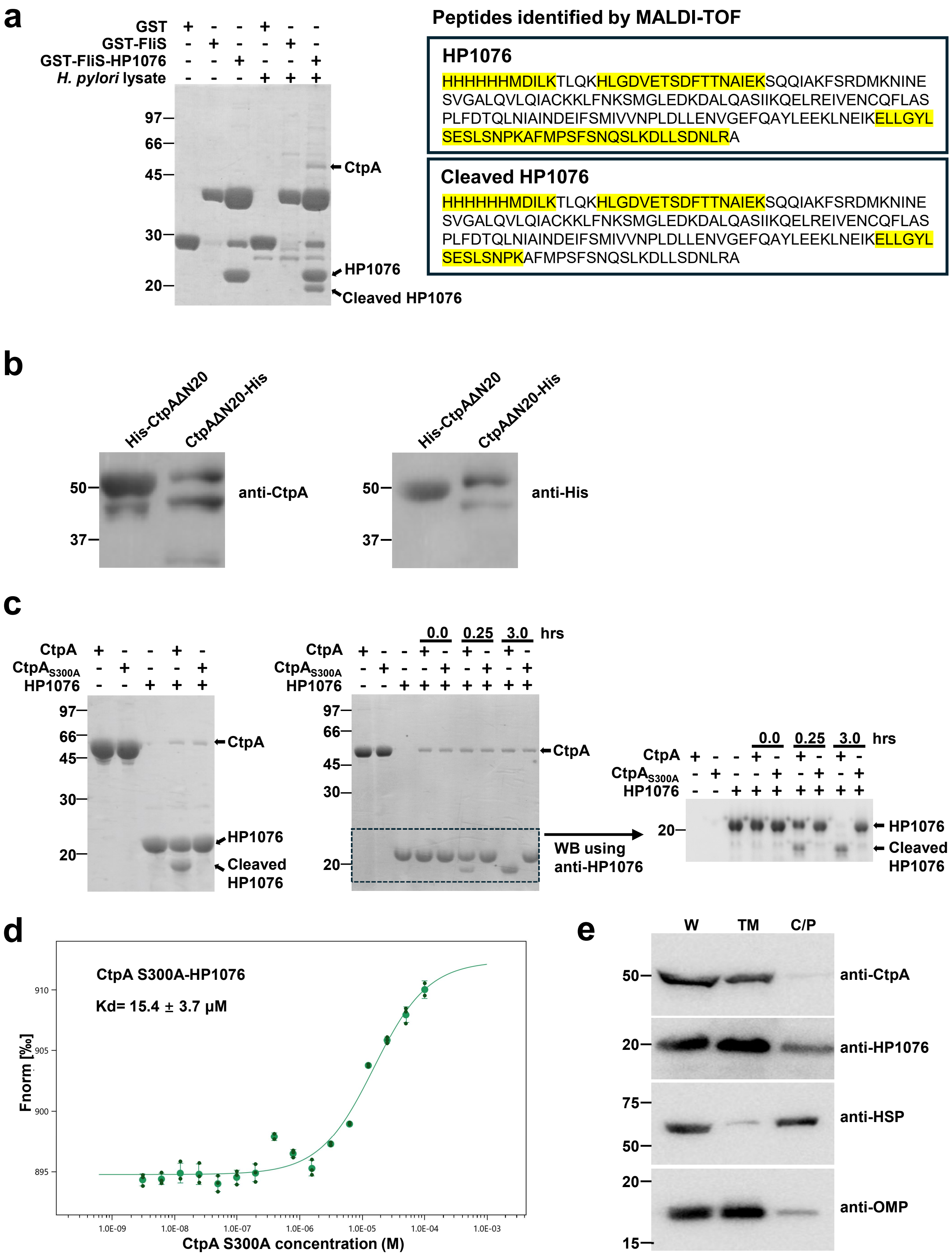

Supplementary Figure 1. (Continues on next page)

f

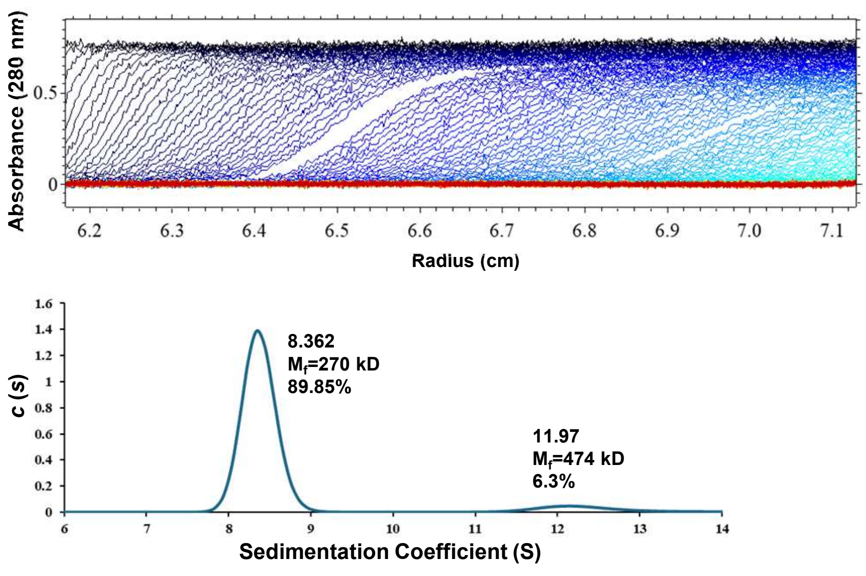

g

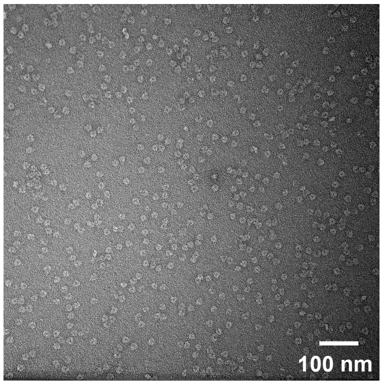

TEM image of CtpA particles

Supplementary Figure 1 continued. Identification of HP1076 as the putative substrate of CtpA and the preliminary characterisation of CtpA. **(a)** Pull-down assay to identify interaction partners of FliS or HP1076. GST was included as a negative control. Extra bands and their identities are indicated. Peptides of HP1076 and cleaved HP1076, identified by MALDI-TOF, are mapped on the HP1076 sequence. **(b)** Purified His-CtpAΔN20 and CtpAΔN20-His were analyzed by immunoblotting with anti-CtpA and anti-His antibodies. The anti-CtpA blot confirmed that CtpAΔN20 was present but underwent cleavage during purification. The absence of signal with anti-His in the N-terminally tagged construct, together with the preserved signal in the C-terminally tagged construct, indicates that the cleavage occurred at the N-terminus of CtpA. **(c)** *In vitro* activity assay of wild-type CtpA and the $CtpA_{S300A}$ mutant (left). The proteolytic activity of CtpA and the importance of Ser-300 were further confirmed by a time-dependent activity assay (middle). The cleavage of HP1076 was confirmed by western blot (right). **(d)** Microscale thermophoresis assay showing the interaction between HP1076 and the $CtpA_{S300A}$ mutant, with error bars showing standard deviation (n=3 independent experiments). **(e)** *H. pylori* cells were fractionated into whole cell (W), total membrane (TM) and cytosolic/periplasmic (C/P) fractions, followed by immunoblotting to detect the corresponding proteins. HSP and OMP were used as the controls for the C/P and TM fractions, respectively. **(f)** Sedimentation velocity analysis of CtpA. The moving boundaries were recorded every 3 min. The calculated $c(s)$ distribution was plotted as a function of the sedimentation coefficient. **(g)** CtpA was negatively stained and visualised via TEM. A scale bar of 100 nm is indicated.

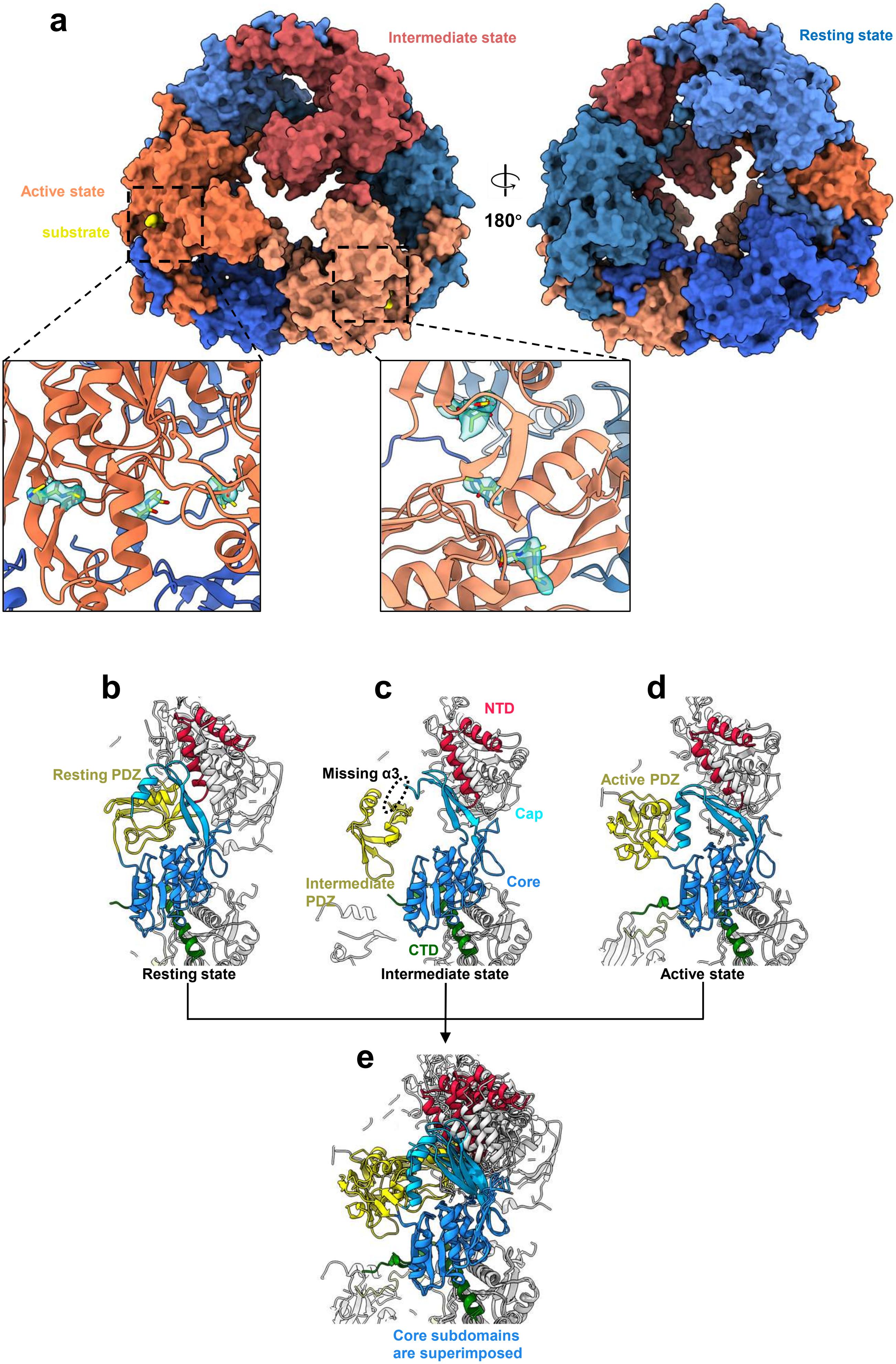
a
Intermediate state
Resting state
Active state
substrate
180°
b
c
d
NTD
Resting PDZ
Missing α3
Cap
Active PDZ
Intermediate PDZ
Core
CTD
Resting state
Intermediate state
Active state
e
Core subdomains are superimposed

Supplementary Figure 2. Atomic model of the wild-type CtpA crystal structure. (a) Crystal structure of wild-type *Hp*CtpA in surface view. The three resting subunits at one face are coloured in shades of blue, and the corresponding ribbon model is depicted in **(b)**. The intermediate subunit is coloured in red, and the corresponding ribbon model is depicted in **(c)**. The missing α3 is indicated by the dotted circle. The two active subunits with substrate binding are coloured in salmon and orange respectively, and the corresponding ribbon model is depicted in **(d)**. The substrates are coloured in yellow in (a). The electron densities of the substrates are shown in the enlarged windows. The protease core subdomains of (b), (c) and (d) are superimposed in **(e)**.

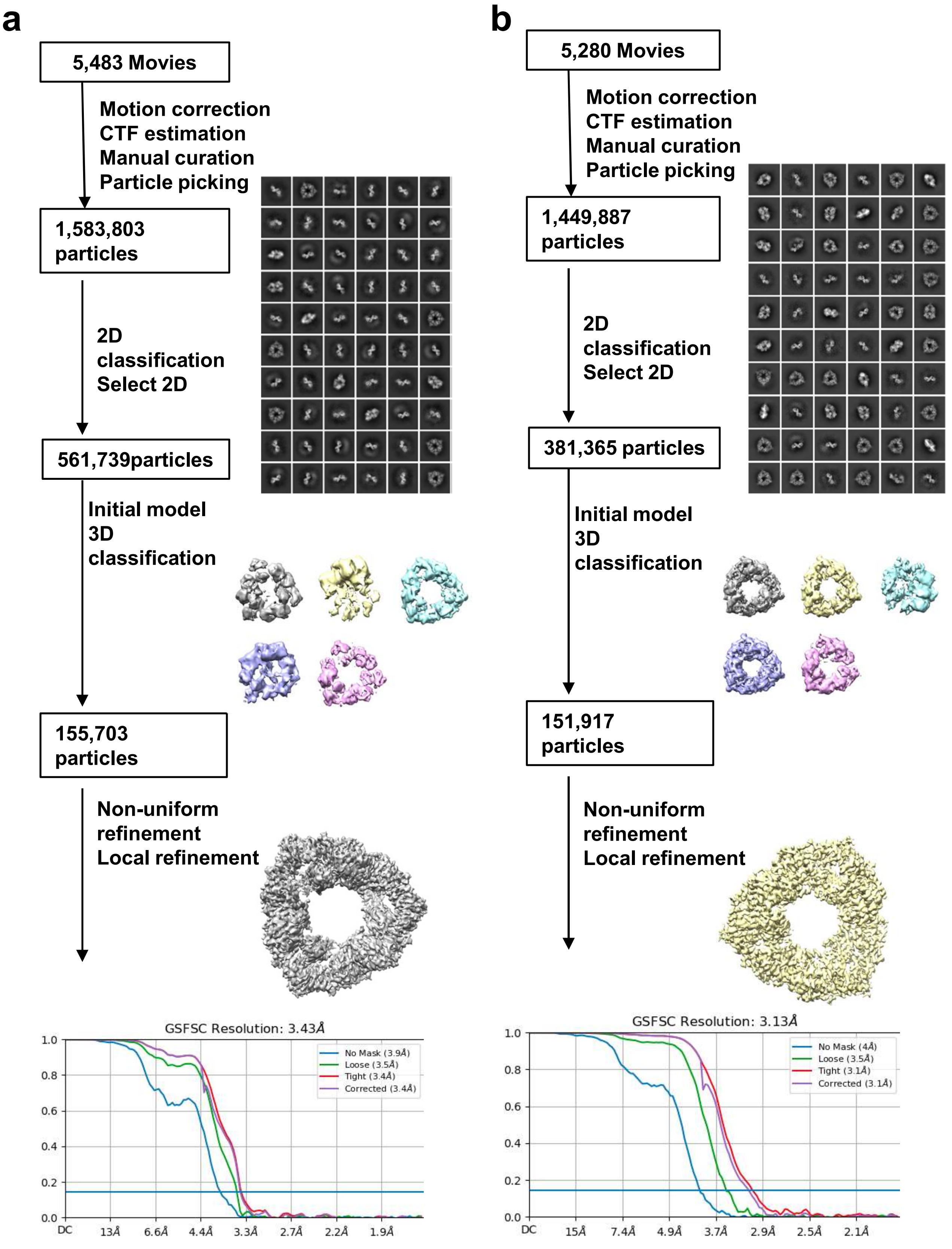

Supplementary Figure 3. Workflow of cryo-EM data processing, representative micrographs, 2D class averages and the Fourier shell correlation curve. (a) Workflow for the single-particle analysis of wild-type CtpA. **(b)** Workflow for the single-particle analysis of $\text{CtpA}_{\text{S300A/K325A/Q329A}}$.

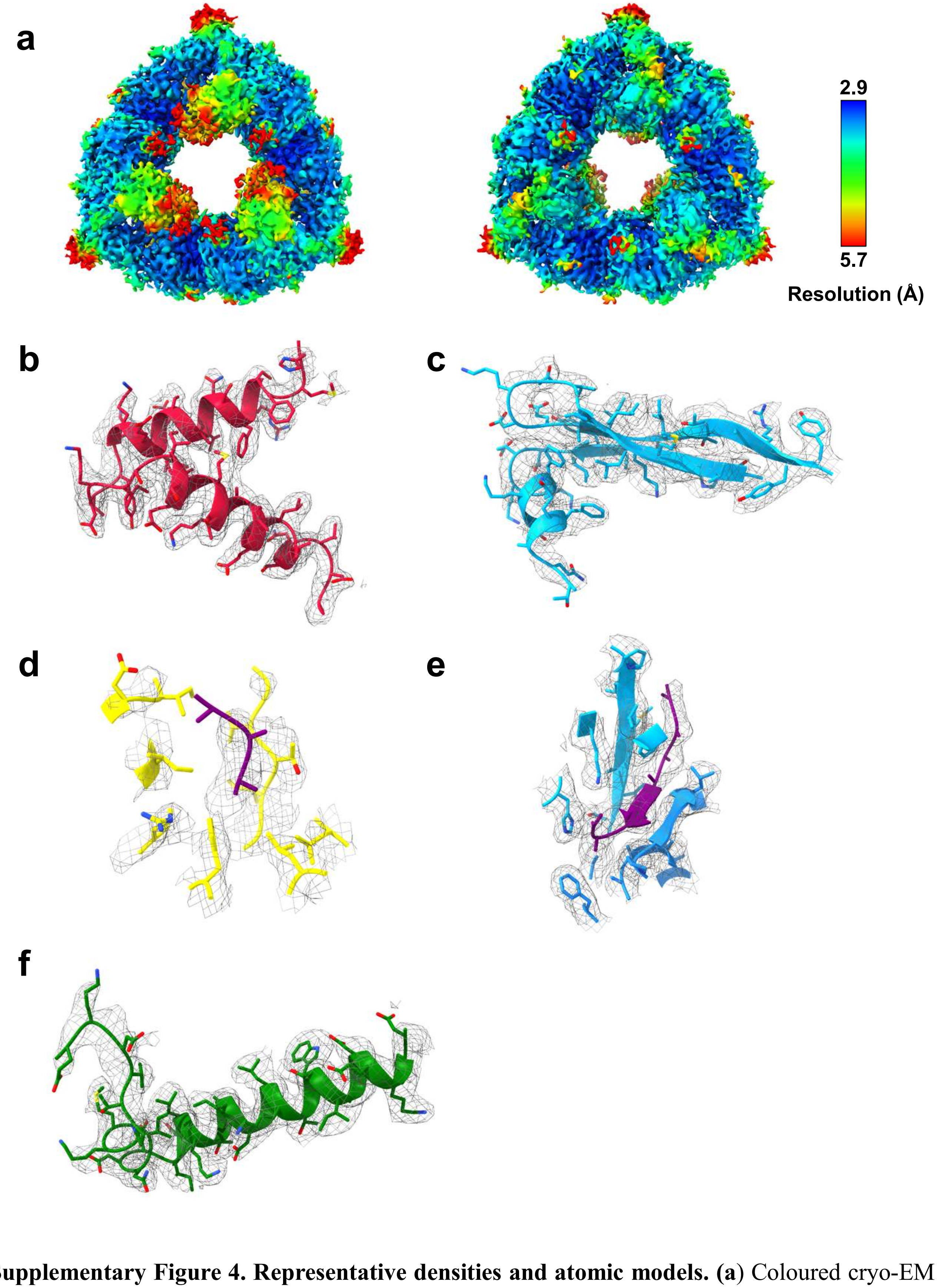

Supplementary Figure 4. Representative densities and atomic models. (**a**) Coloured cryo-EM map of $CtpA_{S300A/K325A/Q329A}$ with local resolutions ranging from 2.9 to 5.7 Å. **(b–f)** Representative densities and atomic models of different domains.

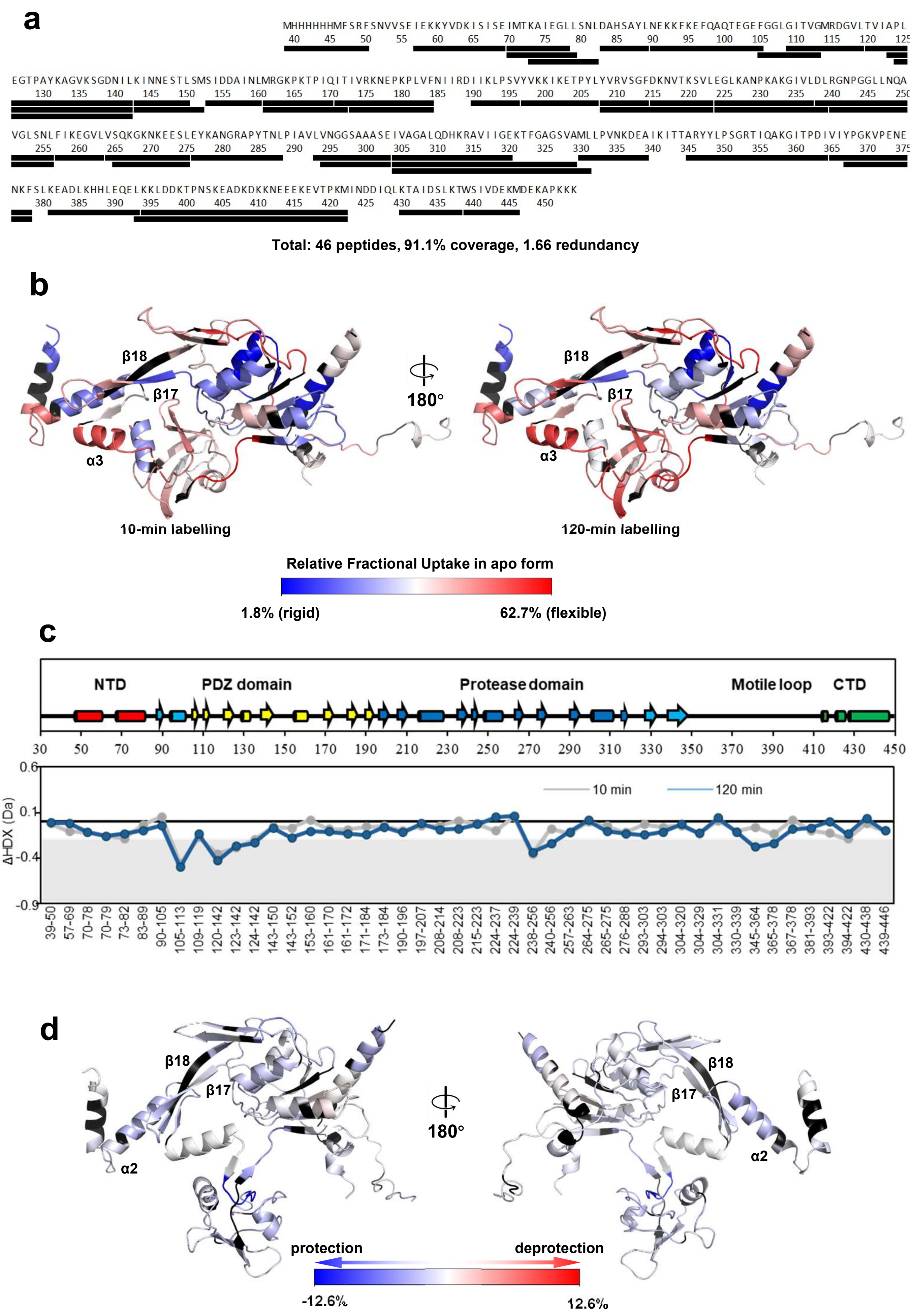

a
MHHHHHHMFSRFSNVVSEIEKKYVDKISISEIMTKAIEGLLSNLDAHSAYLNEKKFKEFQAQTEGEFGGLGITVGMRDGVLTVIAPL
EGTPAYKAGVKSGDNILKINNESTLSMSIDDAINLMRGKPKTPIQITIVRKNEPKPLVFNIIRDIIKLPSVYVKKIKETPYLYVRVSGFDKNVTKSVLEGLKANPKAKGIVLDLRGNPGGLLNQA
VGLSNLFIKEGVLVSQKGKNKEESLEYKANGRAPYTNLPIAVLVNGGSAAASEIVAGALQDHKRAVIIGEKTFGAGSVAMLLPVNKDEAIKITTARYYLPSGRTIQAKGITPDIVIYPGKVPENE
NKFSLKEADLKHHLEQELKKLDDKTPNSKEADKDKKNEEEKEVTPKMINDDIQLKTAIDSLKTWSIVDEKMDEKAPKKK
Total: 46 peptides, 91.1% coverage, 1.66 redundancy
b
β18
β17
α3
180°
10-min labelling
120-min labelling
Relative Fractional Uptake in apo form
1.8% (rigid)
62.7% (flexible)
c
NTD
PDZ domain
Protease domain
Motile loop
CTD
10 min
120 min
ΔHDX (Da)
d
β18
β17
α2
180°
protection
deprotection
-12.6%
12.6%

Supplementary Figure 5. HDX-MS analysis of CtpA. (a) Sequence coverage of $CtpA_{S300A/K325A/Q329A}$. **(b)** Relative fractional deuterium uptake at 10 and 120 min is mapped on the resting form of the CtpA structure model. **(c)** The differences in deuterium uptake of all the peptides identified from CtpA (ΔHDX, CtpA-HP1076 in complex state minus CtpA apo state) at 10- and 120-min time intervals, with the secondary structures shown at the top. Significant differences are shaded in grey. **(d)** The summed HDX difference of 10- and 120-min labelling is mapped on the active form of the CtpA structure model.

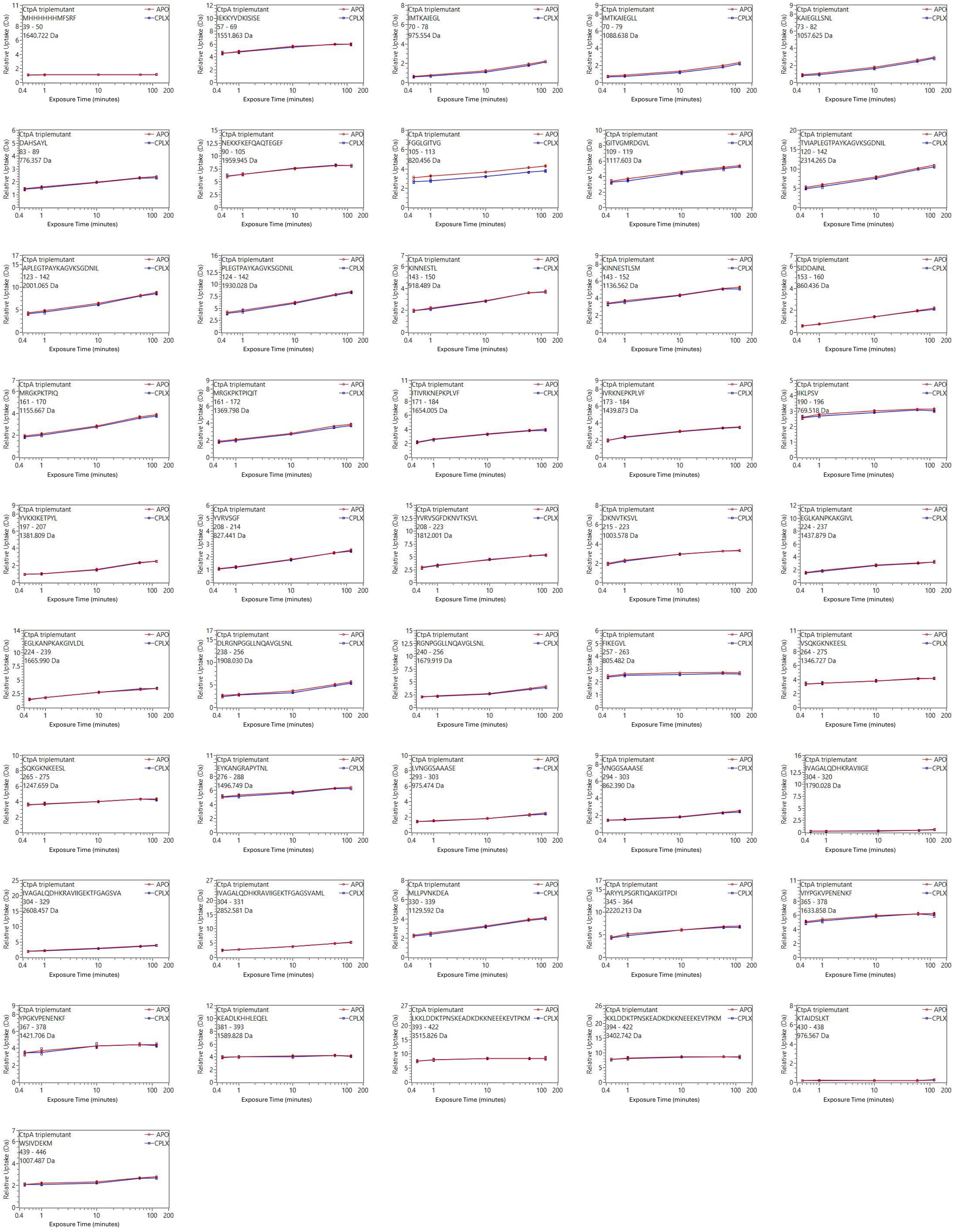

Supplementary Figure 6. HDX-MS time courses for all peptides analysed in apo CtpA$_{TM}$ and CtpA$_{TM}$ in complex with HP1076. HDX measurements were taken at 0, 0.5, 1, 10, 60, and 120 minutes, with three replicates per time point.

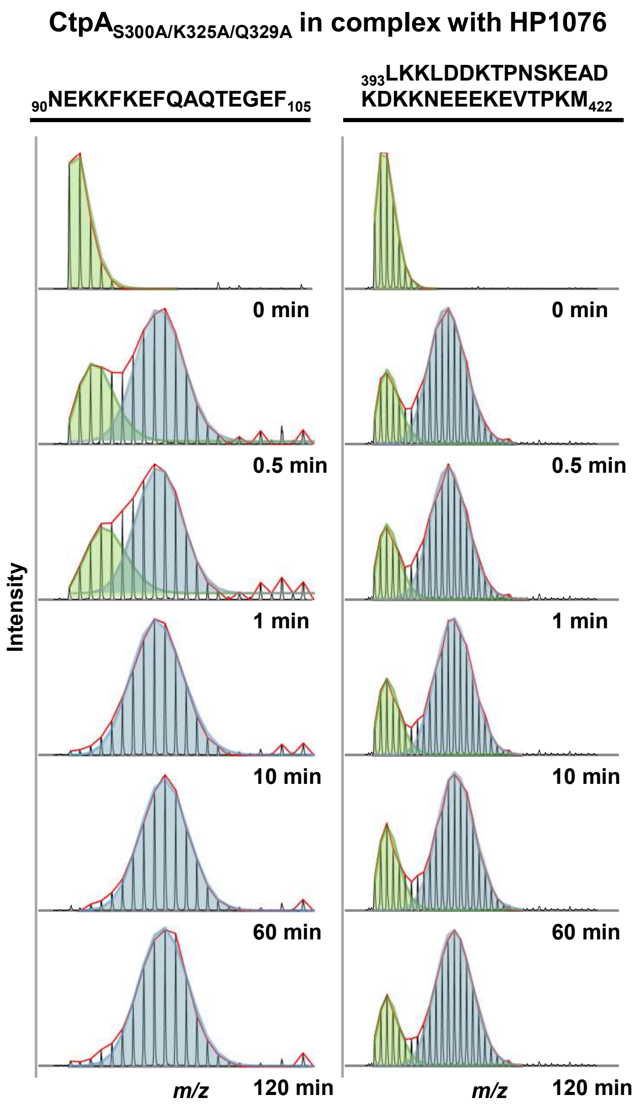

Supplementary Figure 7. Stacked spectral plots of peptides covering residues 90–105 and 393–422 from CtpA$_{S300A/K325A/Q329A}$ in complex with HP1076. The HDX intervals were 0, 0.5, 1, 10, 60 and 120 min.

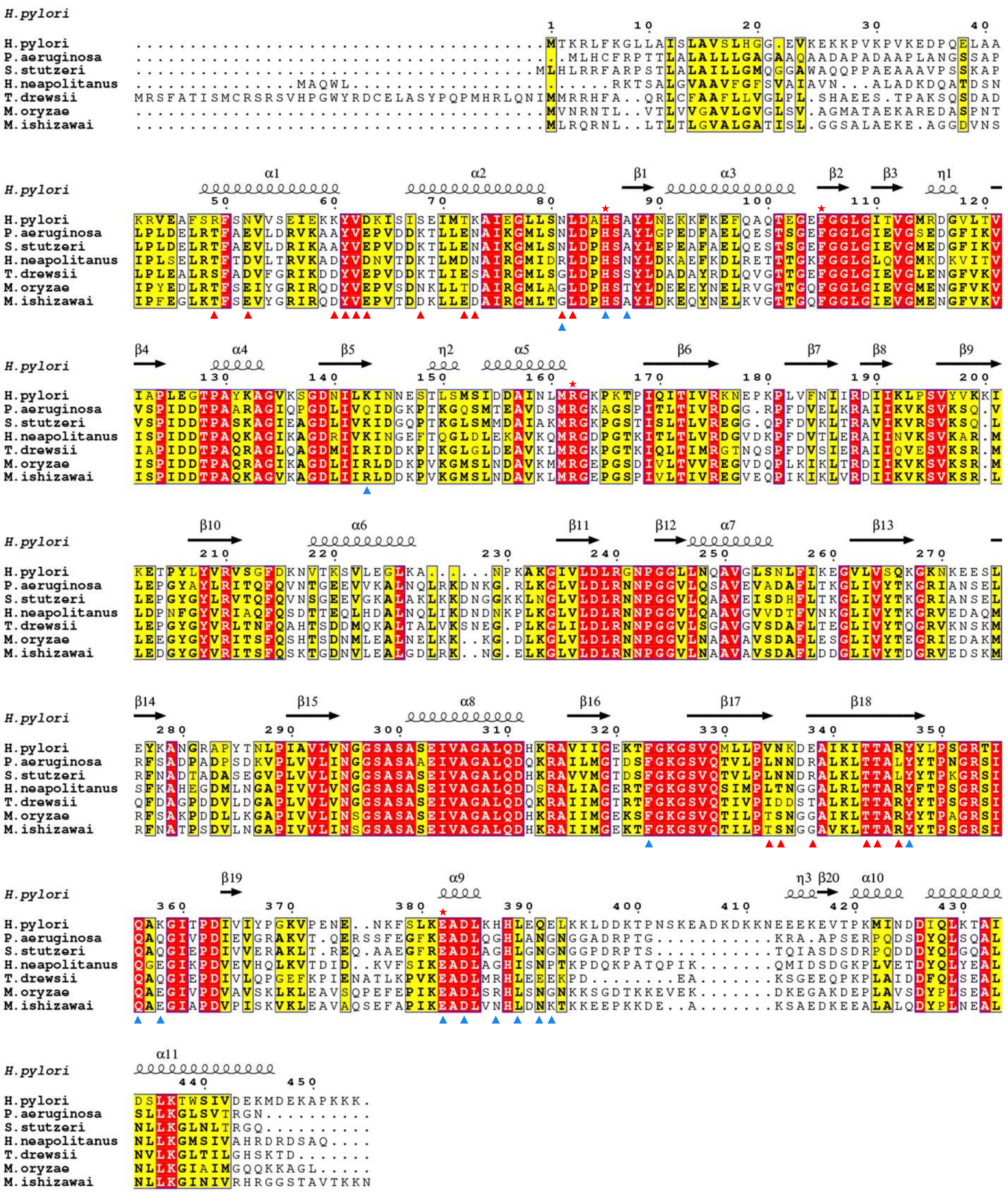

Supplementary Figure 8. Sequence alignment of CtpA from different strains. The secondary structures of *Hp*CtpA are labelled above the sequences. The conserved His-85, Phe-105, Arg-162 and Glu-382 are marked with asterisks. The residues involved in the interactions within the dynamic unit are marked with red triangles. The residues involved in the interactions between ML1 and S_a/S_r are marked with blue triangles. ESPript 3 (Easy Sequencing in PostScript) software (52) was used to create the visual representation of the sequence alignment.

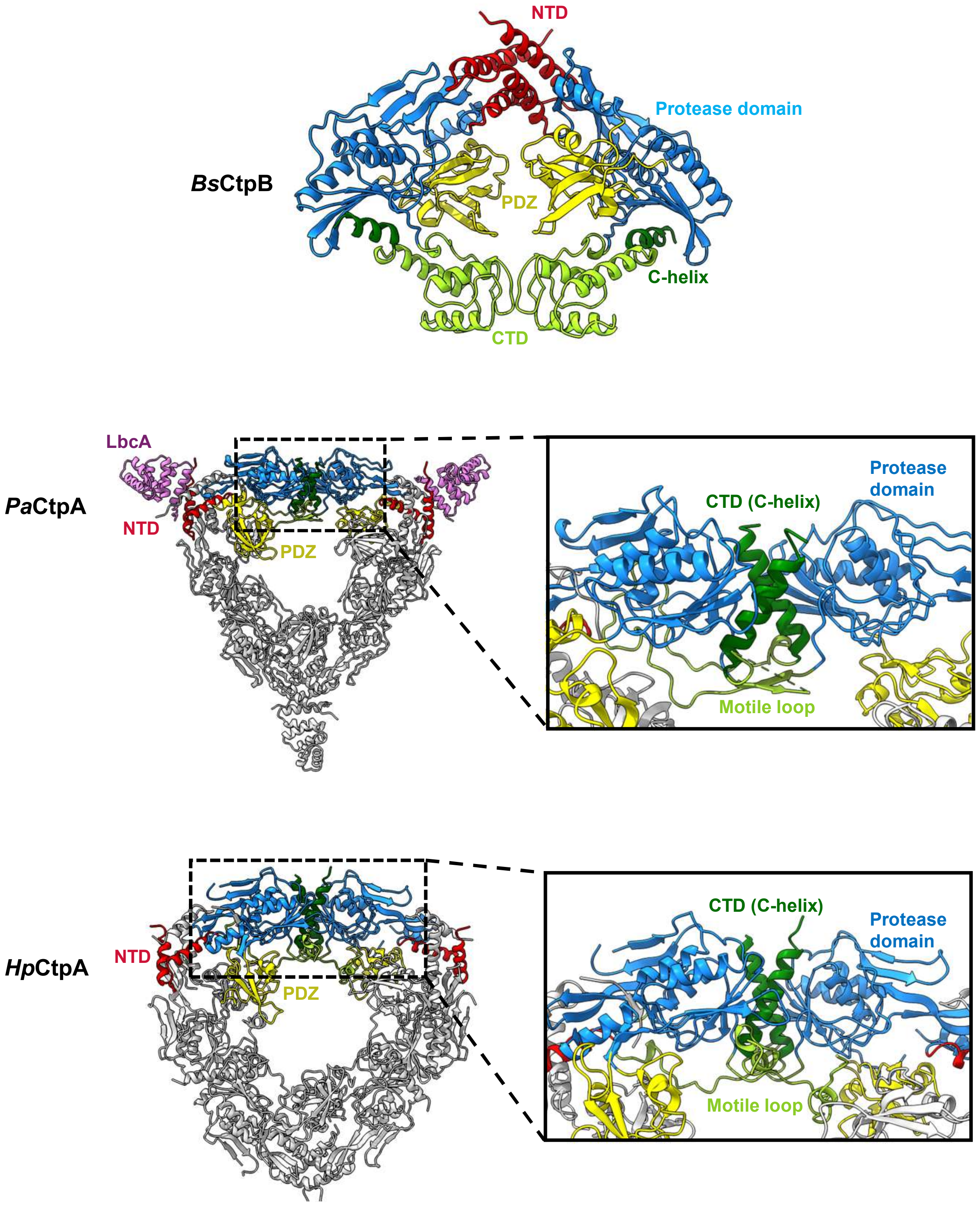

Supplementary Figure 9. Structural comparison of the C-terminal dimerization interfaces in *Bs*CtpB, *Pa*CtpA and *Hp*CtpA. Ribbon representations of the *Bs*CtpB dimer (based on 4C2E; see reference 9), *Pa*CtpA hexamer in complex with LbcA(based on 8SXH; see reference 16), and *Hp*CtpA hexamer are shown. A C-dimer in *Pa*CtpA or *Hp*CtpA is highlighted. The NTD, PDZ domain, protease domain, and C-helix of each CTP are colored in red, yellow, and green, respectively. The CTD of *Bs*CtpB and the motile loops of *Pa*CtpA and *Hp*CtpA are colored in light green. The adaptor protein of *Pa*CtpA LbcA is colored in purple.

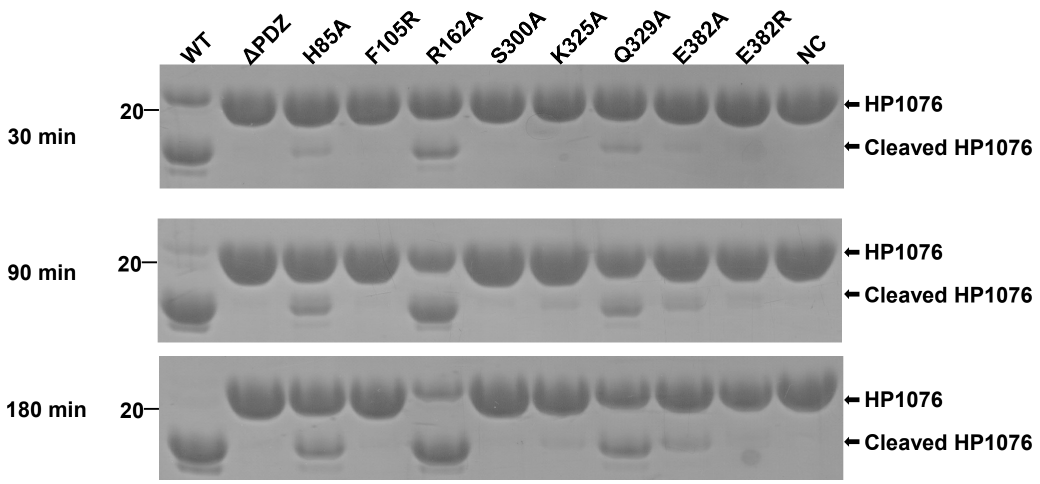

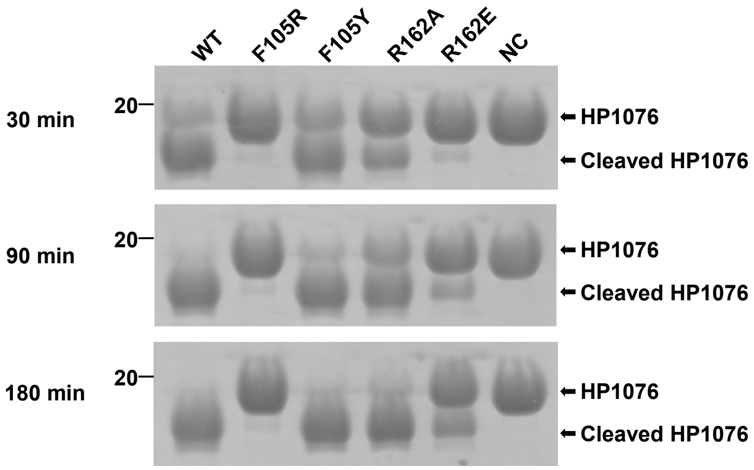

Supplementary Figure 10. **HP1076 cleavage catalysed by wild-type CtpA and various CtpA mutants, monitored using SDS-PAGE assays.** HP1076 was mixed with wild-type CtpA or CtpA mutants at a molar ratio of 10:1 and incubated at room temperature for 30, 90 and 180 min. A negative control (NC) was included without adding CtpA.

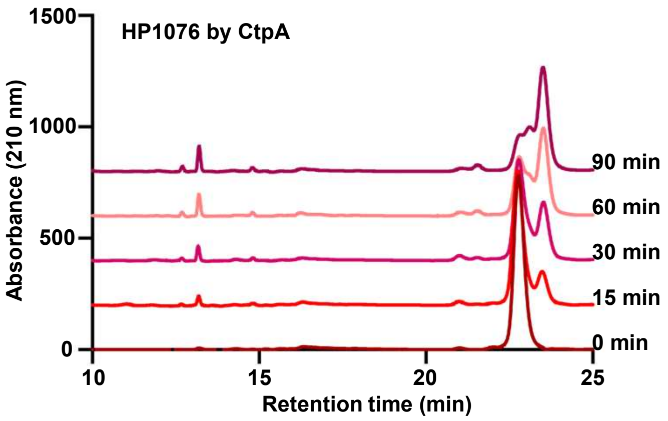

Supplementary Figure 11. HPLC profiles of HP1076 degraded by CtpA (left) or trypsin (right) for 0, 15, 30, 60, 90 min. 50 μg of HP1076 was incubated with either 5 μg of CtpA or 3 μg of trypsin. After quenching the reactions using guanidine hydrochloride, the proteolysis was examined by reverse-phase HPLC.

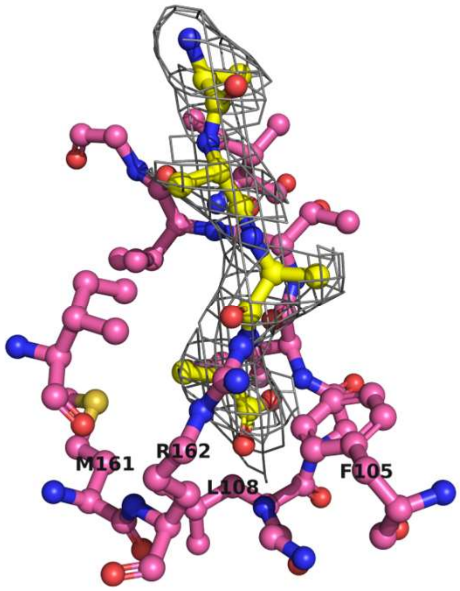

Supplementary Figure 12. Stereo view on the architecture of the ligand binding site in the PDZ domain. The ligand is displayed as an atomic model, with carbon atoms colored in yellow. The corresponding electron density map is overlaid as a mesh representation. Surrounding residues are represented in atomic models with carbon atoms colored in pink.

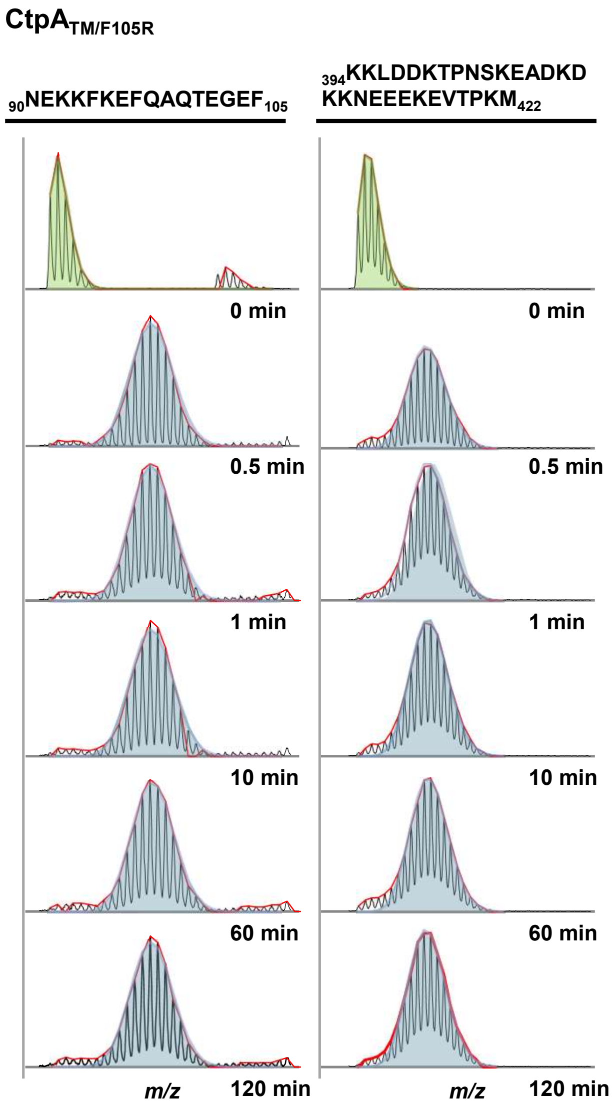

Supplementary Figure 13. Stacked spectra plots of peptides covering residues 90-113 and 394-422 from CtpA$_{TM/F105R}$. The HDX intervals were 0, 0.5, 1, 10, 60 and 120 min. No bimodal distribution is observed.

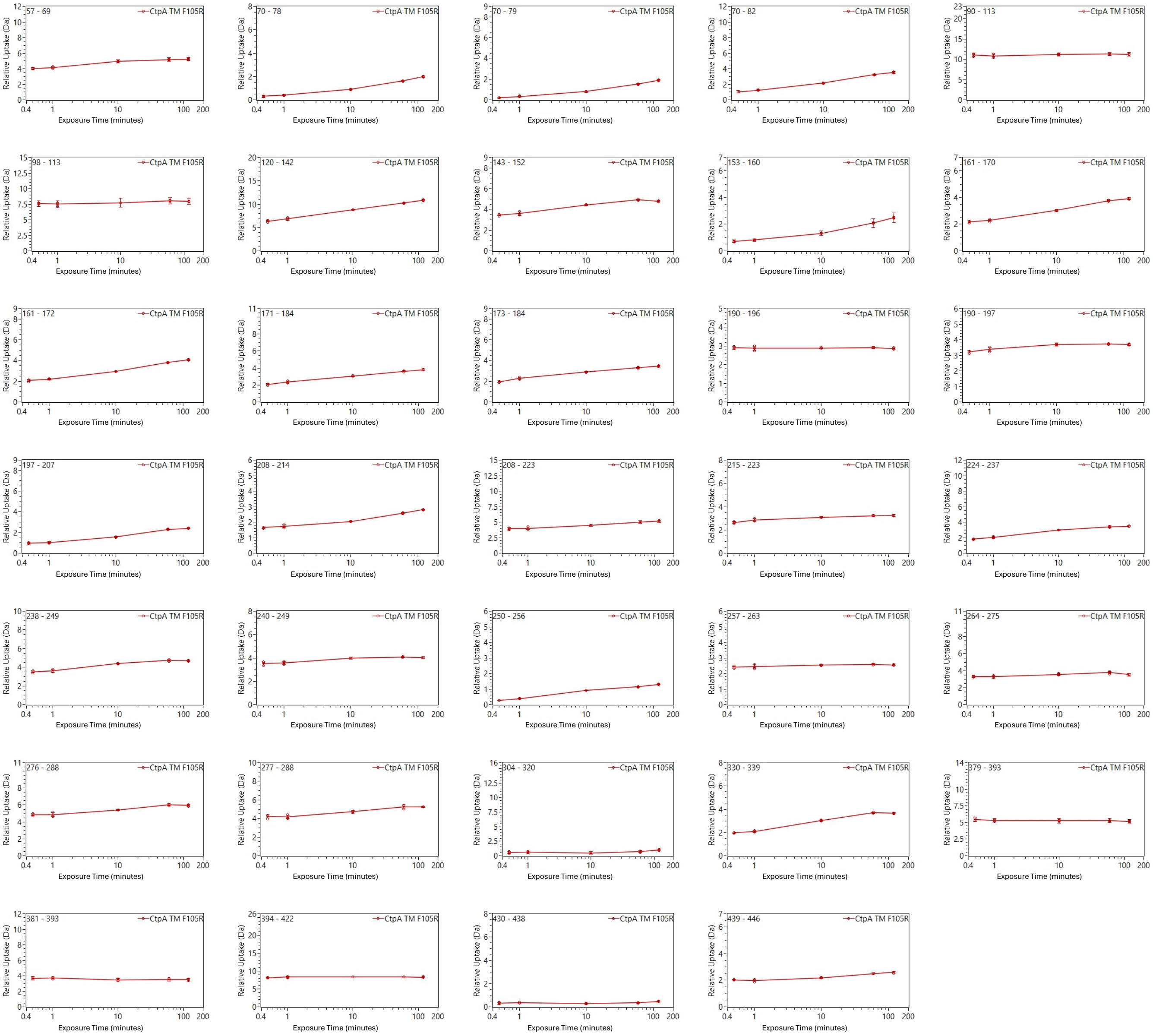

Supplementary Figure 14. HDX-MS time courses for all peptides analysed in $\mathbf{CtpA_{TM/F105R}}$. HDX measurements were taken at 0, 0.5, 1, 10, 60, and 120 minutes, with three replicates per time point.

Supplementary Fig. 1a

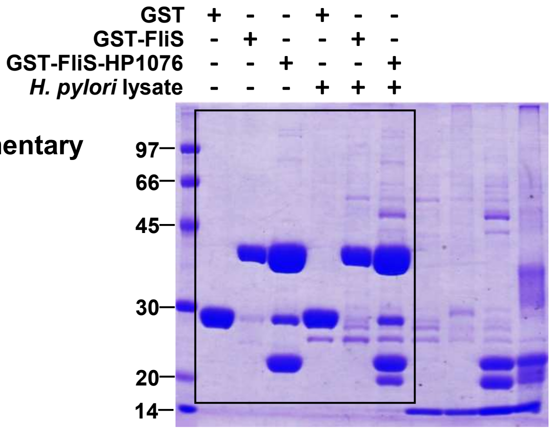

Supplementary Fig. 1b

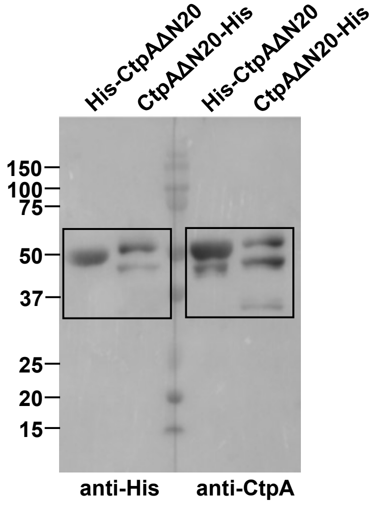

Supplementary Fig. 1c

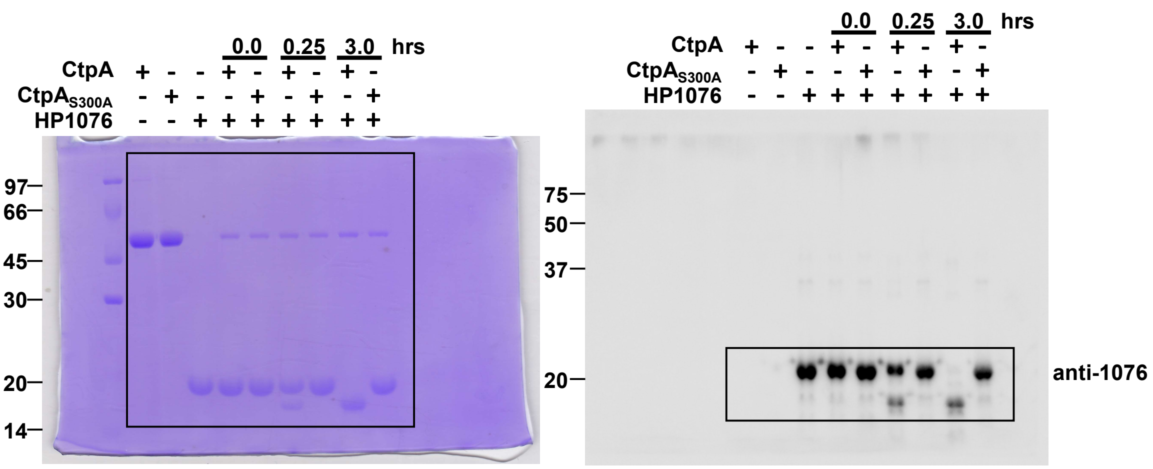

Supplementary Fig. 1e

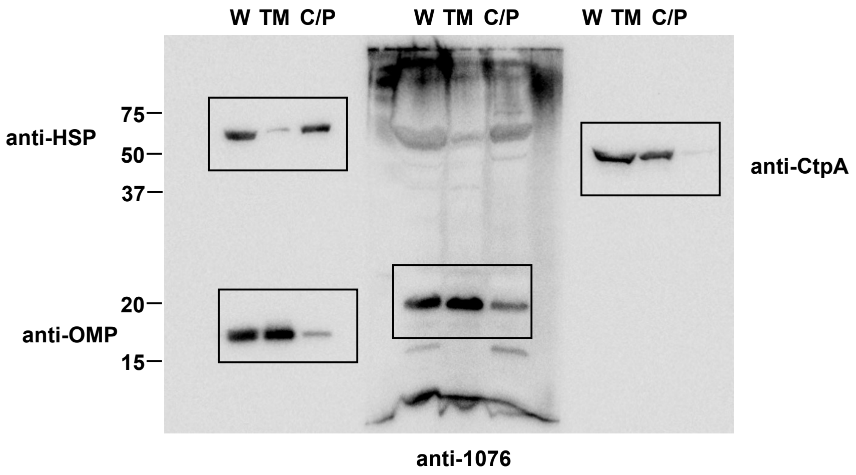

Supplementary Fig. 10

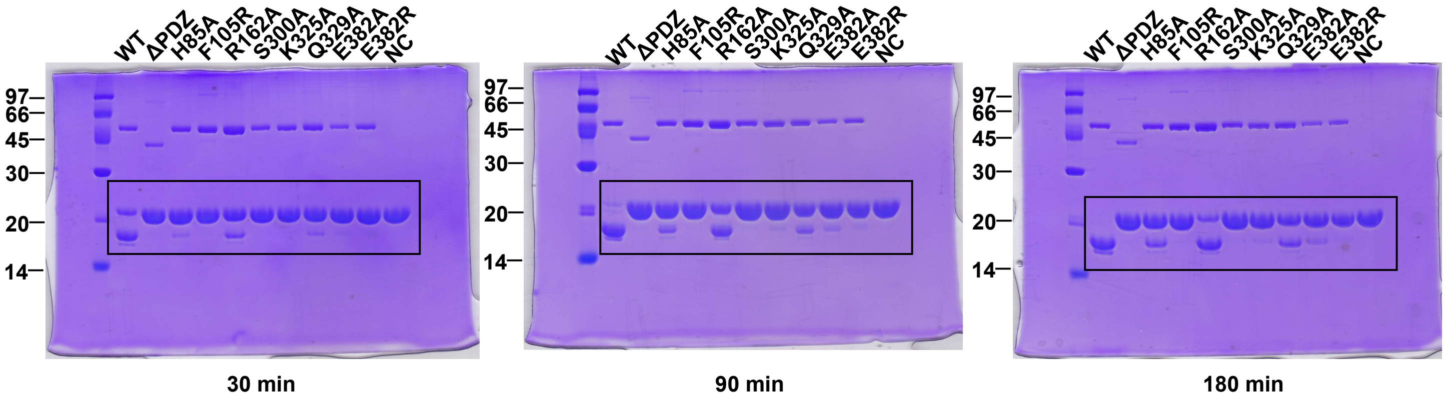

Supplementary Figure 15. (Continues on next page)

Supplementary Fig. 10

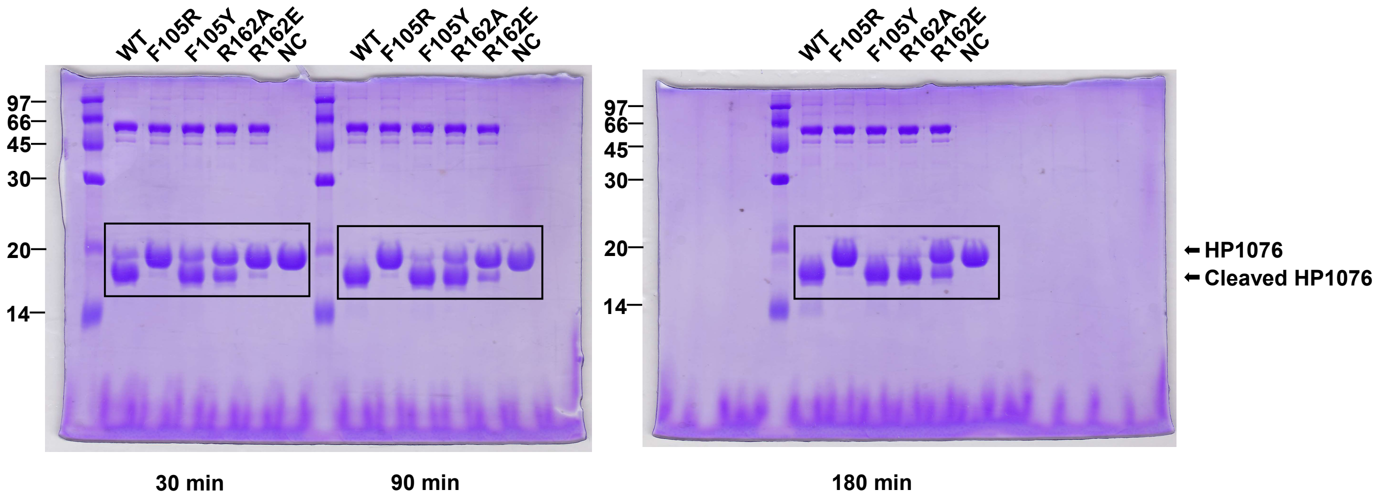

Supplementary Figure 15 continued. Uncropped gel images and western blots.

Black boxes indicate the approximate areas used in corresponding figures.

Supplementary Table 1. Summary of the details for the HDX experiments

Data set	$CtpA_{TM}$	$CtpA_{TM}$ bound with HP1076	$CtpA_{TM/F105R}$
HDX reaction details	20 mM Tris-HCl, 150 mM NaCl in 95% D2O, pH 7.5, RT		
HDX time course (min)	0.5, 1, 10, 60, 120		
Number of peptides	46	46	34
Sequence coverage	91.1%	91.1%	73.5%
Average peptide length /Redundancy	13/1.66	13/1.66	11/1.39
Replicates (biological or technical)	3	3	3
Repeatability	0.095 Da	0.095 Da	0.107 Da
Significant differences in HDX (ΔHDX)	NA	0.19 Da (95% confidence interval)	NA

Supplementary Table 2. Interactions within the dynamic unit

Hydrogen bond		Salt bridge	
Subunit 1	Subunit 2	Subunit 1	Subunit 2
Arg49@NE	Glu338@OE2	Arg49@NE	Glu338@OE1
Arg49@NH1	Glu338@OE2	Arg49@NE	Glu338@OE2
Arg49@NH2	Ser68@O	Arg49@NH1	Glu338@OE1
Arg49@NH2	Thr72@OG1	Arg49@NH1	Glu338@OE2
Arg49@O	Asn335@ND2	Asp63@OD1	Arg346@NH1
Asn52@ND2	Val334@O	Asp63@OD2	Arg346@NH1
Asn52@ND2	Asn335@OD1	Asp63@OD1	Arg346@NH2
Tyr61@OH	Leu82@O	Asp63@OD2	Arg346@NH2
Val62@N	Thr343@O	Arg346@NH1	Asp63@OD1
Asp63@OD2	Arg346@NH1		
Asp63@OD1	Arg346@NH2		
Thr72@OG1	Arg49@NH1		
Lys73@NZ	Asn81@O		
Asn81@O	Lys73@NZ		
ASN81@OD1	Lys73@NZ		
Leu82@O	Tyr61@OH		
Thr343@O	Val62@N		
Thr344@OG1	Lys60@O		
Arg346@NH1	Asp63@OD1		

Supplementary Table 3. Interactions between ML1 and S_a/S_r

**Hydrogen bond**		**Salt bridge**	
ML1	S_a	ML1	S_a
Glu382@OE2	Gln356@NE2	Glu382@OE1	His85@NE2
Glu382@N	Phe323@O		
His387@O	Ala84@N		
His387@NE2	Asn81@OD1		
Leu389@O	Gln356@NE2		
Gln391@OE1	Lys358@NZ		
Glu392@OE2	Tyr348@OH		
ML1	S_r	ML1	S_r
NA	NA	Asp384@OD2	Lys143@NZ

Supplementary references

52. Robert, X. & Gouet, P. Deciphering key features in protein structures with the new ENDscript server. *Nucl. Acids Res*. **42**(W1), W320-W324 (2014). https://doi.org/10.1093/nar/gku316.
