# Supplementary material for: Structures of Helicobacter pylori C-terminal protease CtpA reveal a new mode of self-contained proteolytic processing
Source: Commun Biol. 2025 Nov 22;8:1828. doi: 10.1038/s42003-025-09175-5 (PMC12749690; doi:10.1038/s42003-025-09175-5)
Supplement: Supplementary file 3 — Description of Additional Supplementary Materials [file 42003_2025_9175_MOESM3_ESM.pdf]

Description of Additional Supplementary Files

File name: Supplementary Data 1-2

Description: The numerical source data underlying the graphs presented in this study
